# Arthroscopic Internal Drainage with Cyst Wall Resection and Arthroscopic Internal Drainage with Cyst Wall Preservation to Treat Unicameral Popliteal Cysts: A Retrospective Case–Control Study

**DOI:** 10.1111/os.12917

**Published:** 2021-05-04

**Authors:** Min Zhang, Hao Li, Hao‐hao Wang, Gang Xi, Ya‐kun Li, Bin Zhao

**Affiliations:** ^1^ Department of Orthopedics The Second Hospital of Shanxi Medical University Taiyuan China

**Keywords:** Arthroscopy, Cyst wall, Knee, Minimally invasive treatment, Popliteal cysts

## Abstract

**Objective:**

To compare the clinical efficacy and safety of arthroscopic internal drainage for the treatment of unicameral popliteal cysts with or without cyst wall resection.

**Methods:**

This was a retrospective case–control study of 73 patients diagnosed with unicameral popliteal cysts from January 2012 to January 2019 who received arthroscopic treatment. The study included 38 cases with cyst wall resection (CWR group) and 35 cases with cyst wall preservation (CWP group). The CWR group consisted of 14 men and 24 women with an average age of 51.8 years, while the CWP group consisted of 13 men and 22 women with an average age of 52.0 years. All patients were examined for intra‐articular lesions and communicating ports by magnetic resonance imaging (MRI) prior to surgery, and recurrence of cysts was evaluated at the last follow‐up examination. Rauschning and Lindgren grade (R–L grade) and Lysholm score were used to evaluate clinical outcomes. In addition, operation time and complications were recorded.

**Results:**

The average length of follow‐up was 24.2 months (range, 16 to 32 months). There were no considerable differences in age, gender, cyst size, Lysholm score, R–L grade and concomitant intra‐articular cases between the CWR group and CWP group prior to surgery (*P* > 0.05). The last follow‐up MRI scans showed that in the CWR group, the cyst disappeared in 25 cases and shrunk in 13 cases. In the CWP group, the cyst disappeared in 22 cases, shrunk in 12 cases and persisted in one case. There was no obvious difference in recurrence rate between the two groups (0% *vs* 2.9%, *P* = 0.899). At the last follow‐up, there were no differences in the R–L grade (*P* = 0.630) and Lysholm score (88.3 ± 5.6 points *vs* 90.1 ± 3.8 points, *P* = 0.071) between the two groups. Compared with the CWP group, operation time was significantly prolonged in the CWR group (38.3 ± 3.1 min *vs* 58.3 ± 4.4 min, *P* < 0.05). In the CWR group, three cases occurred fluid infiltration under the gastrocnemius muscle, which improved after pressure bandaging and cold compress. In another three cases, hematoma was found. The incidence of complications in the CWR group was markedly higher than that in the CWP group (15.8% *vs* 0%, *P* < 0.05). During the follow‐up period, none of the patients developed serious complications such as neurovascular injury, deep venous thrombosis, or infection.

**Conclusion:**

For unicameral popliteal cysts, arthroscopic internal drainage combined with resection of the cyst wall did not further improve the clinical outcomes or reduce the recurrence rate, while prolonging the operation time and increasing the possibility of complications.

## Graphical abstract

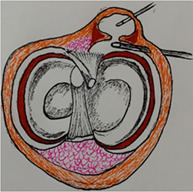

This paper evaluated the necessity of additional removal of the capsule wall after arthroscopic internal drainage in the treatment of popliteal cyst, and concluded that resection of the capsule wall would not produce better outcomes.

## Introduction

Popliteal cyst is a common cystic lesion around the knee joint, most frequently characterized by expansion of the gastrocnemius‐semimembranosus bursa^1^. In 1829, a cystic mass accompanied by extensive effusion in the popliteal fossa of the knee was first reported by Guillaume Dupuytren. In 1877, Baker first described the disease as enlargement of synovial fluid in the popliteal fossa^2^. Baker also reported that popliteal cysts did not exist alone, and were closely related to the internal lesions of the knee joint. In other words, popliteal cysts may be a manifestation of the primary lesion being located inside the joint. In addition, relevant pathological studies^3^, ^4^, ^5^ have found a unidirectional valvular mechanism between the bursa and articular cavity, which is a normal anatomical structure. Taylor and Rana^3^ discovered an existing connection between the medial head of the gastrocnemius muscle and the articular cavity in *post mortem* studies of 50 knee joints. The body of research by Rauschning and Lindgren^4^, ^5^, ^6^, ^7^ detailed the importance of this valve mechanism in knee flexion and extension. From arthroscopic examinations of the knee, Kim *et al*.^8^ described the relationship between the posteromedial capsular fold and popliteal cysts as a “one‐way valve” that may result in an accumulation of effusion. Therefore, most studies support intra‐articular pathology and unidirectional valve interactions as playing a crucial role in the pathogenesis of popliteal cysts.

Popliteal cysts may be divided into primary and secondary cysts based on the different pathogenesis. Secondary popliteal cysts are most common in adults^9^. The extent and severity of symptoms in patients with popliteal cysts depends on the cyst location and the degree of compression on surrounding tissues. In early stages of the disease, symptoms may manifest as distension and stiffness and/or posteromedial pain in the knee^10^, ^11^. Bryan *et al*.^12^ reported that the most common symptoms in patients with popliteal cysts were distension of the popliteal fossa (76%) and posteromedial knee pain (32%). Patients with enlarged cysts are conscious of limitations to knee flexion and extension, which becomes more obvious following activity or fatigue. Popliteal cysts may become firm during full knee extension and soft during flexion, a phenomenon known as “Foucher's Sign”^13^. For patients with mild persistent symptoms, non‐surgical treatments such as observation, nonsteroidal anti‐inflammatory drugs (NSAID), and percutaneous aspiration are recommended^14^. If conservative treatment is ineffective, posterior open surgery or arthroscopic debridement may be performed^15^.

Posterior open surgery has traditionally been used for symptomatic popliteal cysts with ineffective conservation^6^, ^9^. This type of operation not only carries risk of nerve damage, but also requires longer operation times and is more prone to perioperative complications^16^, ^17^, ^18^. In addition, this method only focuses on the cyst itself, and does not consider the intra‐articular pathology and unidirectional valve mechanism, so the cyst may easily recur. Some researchers^19^, ^20^, ^21^ have reported that the recurrence rate after simple open resection was as high as 42% to 63%. With the continuous progress of arthroscopic technology, many scholars have reported that arthroscopic treatment of popliteal cysts achieves satisfactory results. The advantage of arthroscopic treatment of popliteal cysts lies in the treatment of intra‐articular lesions and elimination of the unidirectional flow valve mechanism. Rapid postoperative recovery and low recurrence rates may be additional benefits of arthroscopic treatment^22^, ^23^, ^24^.

Arthroscopy has been widely used in the treatment of popliteal cysts^25^; however, the role and influence of the cyst wall in the development of popliteal cysts remains controversial^26^. It is unclear whether adequate internal drainage to decompress the popliteal cyst under arthroscopy or complete removal of the cyst wall should be standard practice. Arthroscopic internal drainage combined with cyst wall resection *vs* arthroscopic internal drainage only have been used successfully in the treatment of popliteal cysts by different scholars, and their research has already shown some promising results. However, to our knowledge, the majority of studies have independently evaluated the efficacy of internal drainage combined with cyst wall resection or arthroscopic drainage only, but few controlled trials have been designed to compare the outcomes of the two surgical procedures.

In this study, we retrospectively analyzed the outcomes of arthroscopic internal drainage combined with cyst wall resection compared with arthroscopic internal drainage combined with cyst wall preservation in the treatment of unicameral popliteal cysts. The aims of this study were to: (i) compare differences in operative time and clinical efficacy between cyst wall resection and cyst wall preservation; (ii) describe recurrence of popliteal cysts in patients with or without cyst wall preservation and clarify whether additional cyst wall resection is necessary after arthroscopic internal drainage; and (iii) report the incidence of complications between the two groups to compare the safety of the two surgical approaches.

## Materials and Methods

### Inclusion and Exclusion Criteria

#### Study Design

This retrospective case–control study was conducted at the Department of Orthopedics in the Second Affiliated Hospital of Shanxi Medical University from January 2012 to January 2019. The protocol for the study has been approved by the Ethics Committee of the Second Affiliated Hospital of Shanxi Medical University and conforms to the provisions of the Declaration of Helsinki. Written informed consent to participate was obtained from all patients.

#### Inclusion Criteria

The inclusion criteria were: (i) patients with symptomatic unicameral popliteal cysts based on concomitant intra‐articular lesions and communication between the cysts and the articular cavity observed during preoperative MRI; (ii) treatment with arthroscopic internal drainage combined with cyst wall resection; (iii) treatment with arthroscopic internal drainage; (iv) R–L grade, Lysholm score, recurrence, complication were compared; (v) retrospective case–control study.

#### Exclusion Criteria

The exclusion criteria were: (i) previous arthroscopic or open surgery for cysts; (ii) formation of an internal septum leading to a multilocular cyst; (iii) co‐occurring ligament injury, rheumatoid arthritis, infectious arthritis, or gouty arthritis; (iv) co‐occurring diabetes mellitus, hypercoagulable state, or chronic thrombotic disease of lower limbs; (v) postoperative surgery for other diseases; (vi) no complete follow‐up.

### Patient Demographics and Characteristics

Eighty‐eight patients diagnosed with popliteal cysts were retrieved from the medical records system. A total of 76 patients met the inclusion criteria. Thirty‐nine cases were treated by arthroscopic internal drainage combined with cyst wall resection, while 37 cases were treated by arthroscopic internal drainage only. One case in the CWR group received surgical treatment for appendicitis 3 months after the operation, while in the CWP group, one case was lost to follow‐up and one case refused to undergo MRI after the operation. All three patients were excluded from the study. The remaining 73 cases (including 38 cases in the CWR group and 35 cases in the CWP group) were included in the analysis. There were 27 men and 46 women with an average time of onset of 12.8 months (range 6 to 24 months). The popliteal cysts were located in the right knee in 33 cases and in the left knee in 40 cases. All operations were performed by the same senior author.

### Surgical Technique

#### CWP Group

##### Anesthesia and Position

After spinal or general anesthesia, arthroscopic surgery was performed with the patient placed in supine position. A tourniquet applied at the base of the thigh was used to control bleeding and improve vision of the surgical incision.

##### Correcting Concomitant Intra‐Articular Lesions

**Fig 1:**
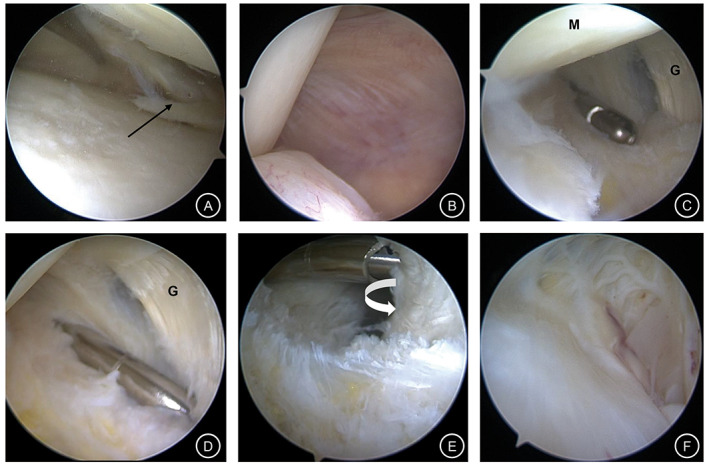
(A) Tear of medial meniscus can be seen under arthroscopy (black arrow). (B) The lens entering the posteromedial compartment after passing through the intercondylar fossa. (C) The medial head of the gastrocnemius muscle exposed after removing the fold of the capsule with a shaver. (D) Internal orifice leading to the cyst was found on the posteromedial side of the medial head of gastrocnemius muscle. (E) The two‐way communication between articular cavity and the cyst (white curved arrow) was restored by enlarging the valve mechanism under arthroscopy. (F) The entire picture of the cyst observed under arthroscopy. (G, medial head of gastrocnemius; M, medial femoral condyle).

Routine anterolateral and anteromedial approaches have been established to explore and treat intra‐articular lesions (such as meniscus tear, cartilage lesion, synovitis, and loose body) (Fig. 1A).

##### Establishment of a Posteromedial Portal

**Fig 2:**
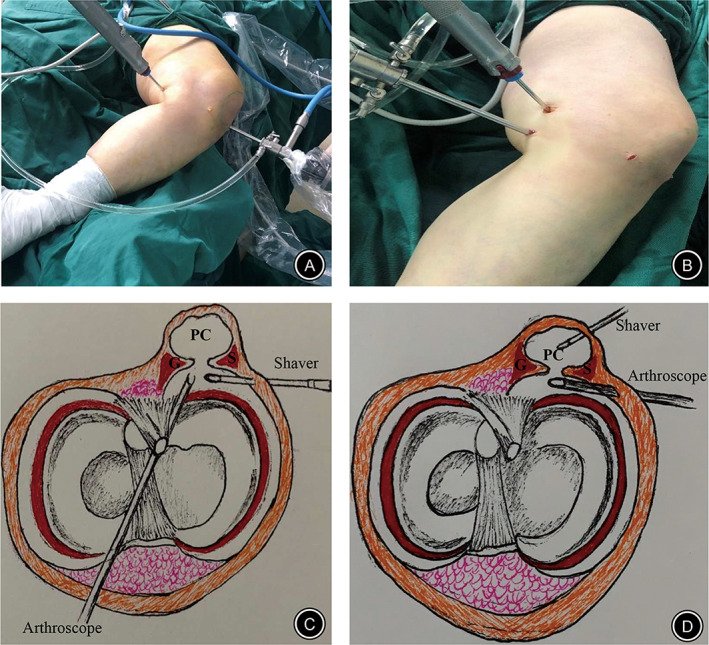
(A) Operation diagram of single posteromedial portal. (B) Operation diagram of double posteromedial portals. (C) Simulation diagram of a single posteromedial portal. (D) Simulation diagram of double posteromedial portals. (G, medial head of gastrocnemius; PC, popliteal cyst; S, semimembranosus tendon).

After treatment of intra‐articular lesions, the lens entered the posteromedial compartment through the space between the posterior cruciate ligament and the posterior horn of the medial meniscus through the intercondylar fossa at 90° flexion of the knee joint (Fig. 1B). The first standard posteromedial (PM) portal was established by light source positioning (Fig. 2A).

##### Internal Drainage to Decompress the Popliteal Cyst

A shaver and probe were used alternately to find the opening fissure of the gastrocnemius‐semimembranous bursa at the articular capsule folds behind the femoral medial condyle (Fig. 1C). After confirmation, we used a shaver to remove a portion of the capsular folds through the PM portal to enlarge the valve opening by at least 5 mm (Figs 1D, 2C). The cyst was then pressed to the surface of the body, where light yellow cystic fluid could be observed emanating from the internal portal confirming that internal drainage was complete (Fig. 1E).

##### Suture and Close

Following surgery, the joint cavity was washed and the incision was closed following conventional procedures with no drainage tube. An elastic bandage was used to apply pressure to the sutured area.

#### CWR Group

The position of patients and surgical methods of cyst drainage under arthroscopy were identical to those of the CWP group.

##### Establishment of a High Posteromedial Portal

After internal drainage, the lens was transferred from the anterolateral portal to the PM portal, and extended from the posteromedial side of the medial head of the gastrocnemius muscle to the interior of the cyst, exposing the smooth inner surface of the capsule wall (Fig. 1F). Furthermore, a second high posteromedial (HPM) portal was established 2–3 cm away from the PM portal (Fig. 2B).

##### Cyst Wall Resection

The PM portal was used to observe the cyst under arthroscopy. The HPM portal was used for the arthroscopic surgery (Fig. 2D). A shaver was placed to change the lens direction under monitoring to complete removal of the entire capsule wall.

##### Suture and Close

Following surgery, the joint cavity was washed and the incision was closed following conventional procedures with no drainage tube. An elastic bandage was used to apply pressure to the sutured area.

### Outcome Measures

The medical charts were reviewed to obtain clinical data, including cyst size, intraoperative findings, duration of surgery. Recurrence and complications were recorded during the follow‐up visit. R–L grade and Lysholm score were used to evaluate the preoperative status and postoperative outcome.

#### Clinical Evaluation

##### Rauschning and Lindgren Grade

Rauschning and Lindgren grade (0–3) is used to evaluate knee joint symptoms in patients with popliteal cysts, according to swelling, pain, instability, limitation of movement, and ability to work and participate in sports. The Rauschning and Lindgren grading is as follows: grade 0 – no swelling or pain, no limitation of range of motion, no instability or weakness, no limitation in work or sports participation; grade 1 – slight swelling and discomfort after strenuous exercise, slight weakness and <1 cm muscular atrophy, negligible limitations to range of motion (<10 degrees), patient is restricted from hard labor and elite sports participation; grade 2 – moderate swelling, pain following moderate exertion, slight or moderate instability and 1–2 cm muscular atrophy, slight limitation to range of motion (10°–20°), patient is restricted from physical work and is permitted limited participation in sports; grade 3 – considerable swelling, severe pain interfering with daily activities, pain at rest, disabling instability, contractures and >2 cm muscular atrophy, limited range of motion (>20°), joint may be nonfunctional due to derangement, no participation in sports^27^. Grade 0 predicted the best results, while grade 3 predicted the worst results.

##### Lysholm Score

The Lysholm Knee Scoring Scale is a patient completed questionnaire commonly used to assess the performance and activity restrictions both before and after arthroscopic knee surgery. It consists of eight items: limpness (three options, score range 0–5), support (three options, score range 0–5), locking (five options, score range 0–15), instability (six options, score range 0–25), pain (six options, score range 0–25), swelling (four options, score range 0–10), stair climbing (four options, score range 0–10), squatting (four options, score range 0–5). The total score is the sum of each response to the eight items. Maximum score is 100 points, which means no symptoms or disability. Scores are categorized as excellent (95–100), good (84–94), fair (65–83), or poor (<64)^28^, ^29^.

#### Radiographic Assessment

Ultrasound or MRI was performed on all patients before surgery to confirm the presence of a popliteal cyst and detect the associated intra‐articular pathology. At the last follow‐up, an MRI scan was performed to determine if the cyst had recurred. Complications were also recorded. The results were expressed as disappearance, shrink, and persistence, of which the persistence was identified as recurrence.

### Statistical Analysis

All statistical analyses were performed using SPSS software (version 26.0, IBM Corporation, Chicago, USA). The continuous variables with normal distribution were displayed as mean ± SD deviation. Independent sample t‐tests were used to compare continuous data between groups. Categorical variables were presented with absolute frequencies (n) and percentages (%). Pearson chi‐square test or a Fisher's exact probability test were used to compare count data. The rank variables were examined by Wilcoxon rank sum test. All statistical tests were two‐sided and were evaluated at the significance level of 5%, a *P*‐value <0.05 was considered statistically significant.

## Results

### Follow‐Up

All patients underwent follow‐up examinations in the outpatient department. The average follow‐up period was 24.2 months (range, 16–32 months). The mean follow‐up times of the CWR group and the CWP group were 24.8 months (range, 15–32 months) and 23.5 months (range, 16–32 months), respectively. The subgroup data were presented in Table 1, which showed no significant difference in the two groups (*P* = 0.247). Symptoms associated with popliteal cysts had disappeared in most patients by the last follow‐up examination.

**TABLE 1:** Demographic characteristics and preoperative data at baseline

	CWR group (n = 38)	CWP group (n = 35)	*P*‐value
Age (years)	51.8 ± 8	52 ± 7.9	0.624
Gender (n, %)			0.979
Male	14 (36.8%)	13 (37.1%)	
Female	24 (63.2%)	22 (62.9%)	
Mean follow‐up (months)	24.8 ± 5.1	23.5 ± 4.5	0.247
Cyst size (mm)	9.1 ± 2.6	8.4 ± 2.1	0.512
Pre‐operative Lysholm score	68.6 ± 4.7	66.9 ± 4.0	0.096
Rauschning and Lindgren grade			0.360
0	0 (0%)	0 (0%)	
I	6 (15.8%)	8 (22.9%)	
II	23 (60.5%)	21 (60%)	
III	9 (23.7%)	6 (17.1%)	
Associated articular pathologies (n, %)			0.990
Degenerative cartilage damage	23 (60.5%)	21 (60%)	
Medial meniscal tear	25 (65.8%)	26 (74.3%)	
Lateral meniscal tear	7 (18.4%)	8 (22.9%)	
Synovitis and synovial hypertrophy	9 (23.7%)	7 (20%)	
Loose body	4 (10.5%)	3 (8.6%)	
Plica syndrome	3 (7.9%)	2 (5.7%)	

### General Results

A total of 73 cases of popliteal cysts was investigated, 38 cases in the CWR group and 35 cases in the CWP group. Baseline characteristics for patients in the two group were listed in Table 1. There were no considerable differences between the two groups in preoperative data including age (*P* = 0.624), gender distribution (*P* = 0.979), cyst size (*P* = 0.512), Lysholm score (*P* = 0.096), and R–L grade (*P* = 0.360).

In all cases, correlative intra‐articular lesions were treated, and the two‐way communication mechanism between the posteromedial compartment and the cyst was restored under arthroscopy. In the CWR group, the cyst wall was completely dissected through the high posteromedial portal combined with internal drainage decompression. In the CWP group, only internal drainage decompression was performed and the cyst wall was preserved.

**Fig 3:**
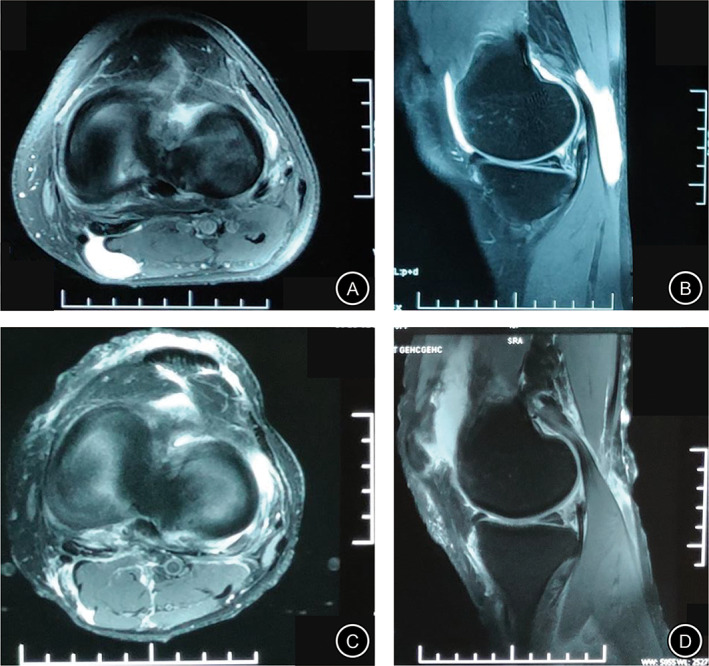
(A & B) Preoperative MRI scan of the axial and sagittal T2 views identifying a popliteal cyst on the left knee of a 55‐year‐old male patient hospitalized for swelling and pain of the left knee for 12.5 months. (C & D) MRI scan of the axial and sagittal T2 views at the last follow‐up examination 22 months after surgery demonstrate disappearance of the popliteal cyst on the left knee.

Intraoperative findings associated with intra‐articular lesions included: medial meniscal tear (n = 51: 25 in the CWR group and 26 in the CWP group) (Fig. 3); cartilage degeneration (n = 44: 23 in the CWR group and 21 in the CWP group); lateral meniscal tears (n = 15: seven in the CWR and eight in the CWP); synovitis (n = 16; nine in the CWR and seven in the CWP); loose body (n = 7: four in the CWR and three in the CWP); plica syndrome (n = 5: three in the CWR and two in the CWP). There was no significant difference between the two groups in the frequency of intra‐articular lesions (*P* = 0.990) (Table 1). The operation time of the CWP group was dramatically shorter than that of the CWR group (38.3 ± 3.1 *vs* 58.3 ± 4.4; *P* < 0.05) (Table 2).

**TABLE 2:** Comparison of operation time, Lysholm score, MRI outcome, and complications between the two groups

	CWR group (n = 38)	CWP group (n = 35)	*P*‐value
Operative time (min)	58.3 ± 4.4	38.3 ± 3.1	0.000
Postoperative Lysholm score	88.3 ± 5.6	90.1 ± 3.8	0.071
Outcome of MR images at the last follow‐up (n, %)			0.899
Disappeared	25 (%)	22 (%)	
Shrunk	13 (%)	12 (%)	
Persisted (recurrence)	0 (%)	1 (%)	
Complications (n, %)	6 (%)	0 (%)	0.026

### Clinical Outcome

#### Rauschning and Lindgren Grade

**Fig 4:**
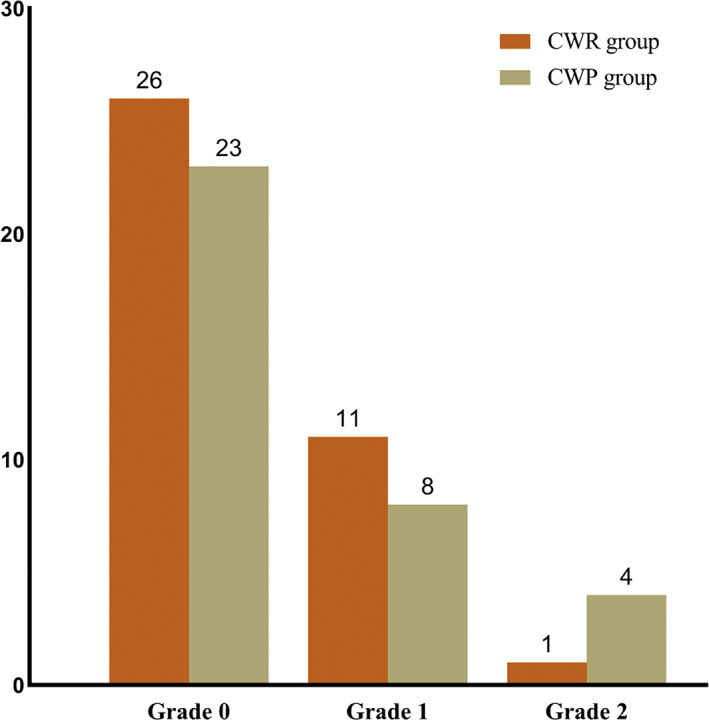
Distributions of Rauschning and Lindgren grade in the two groups at the last follow‐up.

According to the Rauschning and Lindgren grade, the distributions of the CWR group at the final follow‐up were as follows: grade 0 in 26 patients, grade I in 11 patients, and grade II in one patient. The relevant findings in the CWP group at the final follow‐up were: grade 0 in 23 patients, grade I in eight patients, and grade II in four patients (Fig. 4). There was no significant difference between the two groups (*P* = 0.630).

#### Lysholm Score

According to the Lysholm score, the pre‐operative Lysholm score of the CWR group and the CWP group were 68.6 ± 4.7 points and 66.9 ± 4.0 points, respectively. The Lysholm score in the two groups were increased to 88.3 ± 5.6 points and 90.1 ± 3.8 points respectively at the final follow‐up. There was no marked difference in terms of Lysholm score between the two groups (*P* = 0.071) (Table 2).

### Radiographic Improvement

**Fig 5:**
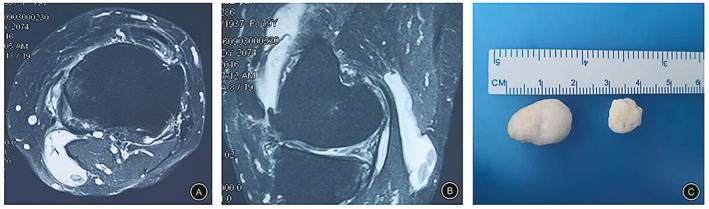
A 63‐year‐old female who presented with distension of the right knee 9 months after resection of a hemangioma in the popliteal fossa of the right lower limb. The patient was treated by knee arthroscopy. (A & B) Preoperative MRI showing a popliteal cyst with loose bodies formation on the right knee. (C) The internal loose bodies were removed when the cyst was treated under arthroscopy, suggesting that the cyst communicated with the articular cavity through the valve opening.

**Fig 6:**
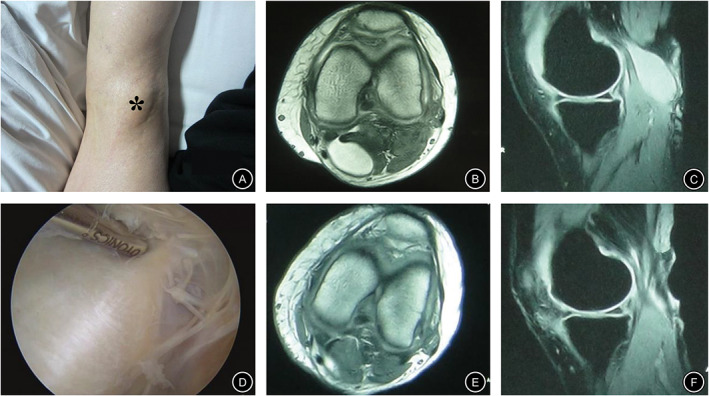
A 55‐year‐old male with posteromedial pain and distension of the right knee for more than 15 months and mild flexion limitation for 5 months. (A) Physical examination showing a cystic mass in the popliteal fossa (*). (B & C) A popliteal cyst identified by MRI on the right knee before surgery. According to R–L grade, the preoperative popliteal cyst was grade 3, but recovered to grade 0 following arthroscopic treatment. (D) Arthroscopic view of the cyst wall. (E & F) No recurrence of the cyst was detected by MRI after 28 months of follow‐up.

A total of 73 patients underwent an MRI examine at the last follow‐up. The results showed that the cyst disappeared in 25 cases (Fig. 5) and shrunk in 13 cases in the CWR group. Comparatively, the cyst disappeared in 22 cases (Fig. 6), shrunk in 12 cases, and persisted in one case in the CWP group, there was no dramatic difference between the two group in terms of recurrence rate (0% *vs* 2.9%, *P* = 0.899) (Table 2).

### Complications

Regarding complications, three cases in the CWR group had persistent swelling and ecchymosis at back of the leg after surgery, which were possibly caused by fluid infiltration under the gastrocnemius muscle, and improved after pressure bandaging and cold compress. No special discomfort was reported until the last follow‐up. In addition, A hematoma was found in three cases in the CWP group. The incidence of complications in the CWR group was markedly higher than that in the CWP group (15.8% *vs* 0%, *P* = 0.026) (Table 2). There were no serious complications such as neurovascular injury, deep venous thrombosis, or infection in both groups.

## Discussion

### Summary of the Major Results of the Study

This study is promising. Some meaningful results have been found. First, patients with multilocular septa were excluded from our study. In fact, we performed microscopical resection of five patients with multilocular cysts, but we did not include them in the study because these patients would generate bias in our baseline groupings. As stated by Ahn *et al*.^30^ and Lie and Ng^31^, complete resection of the cyst is a reasonable choice when there is septum formation in the cyst. Kanekasu *et al*.^32^ removed the cyst wall and synovium of patients with popliteal cysts concerned with rheumatoid arthritis, and concluded that the septum may be related to postoperative recurrence of the cysts. Separation means multiple cavities, each with a complete capsule, which may not be formed at the same stage. Simply expanding the valve connection may lead to inadequate drainage and may cause recurrence. Second, our results show that compared with simple internal drainage, additional removal of the cyst wall will not improve clinical effect and reduce recurrence rate. In this study, the clinical results of the two groups are consistent. Only one patient in the CWP group experienced recurrence. We believe our excellent results with minimal relapse are attributable to expanding the valvular mechanism by at least 5 mm, which is sufficient to restore two‐way circulation^33^. This result further confirms that the valvular mechanism should be corrected during treatment of popliteal cysts. Finally, removal of the cyst wall increases the incidence of complications. Cho^22^ reported that the incidence of complications was 4.5%, of which hematoma was the most common. In 2009, Kp *et al*.^34^ reported that 2 months after arthroscopic cyst wall resection, one patient developed a popliteal artery pseudoaneurysm. Therefore, they suggested that the lateral wall of the cyst should not be planed during cyst wall resection to prevent damage to the popliteal artery. In this study, no serious vascular or nerve injury occurred; however, in three cases, fluid extravasated under the gastrocnemius muscle of the calf and severe swelling was noticeable for several days following surgery. Fortunately, these patients improved after compression and ice were applied.

### Pathogenesis of Popliteal Cyst

Although the etiology of popliteal cyst is still unclear, it is generally believed that intra‐articular pathology plays an indispensable role in the formation of popliteal cyst. Some studies^35^, ^36^ have warned that if related intra‐articular pathology is not corrected, the recurrence rate will be substantial. Johnson *et al*.^11^ described the incidence of different types of intra‐articular lesions in patients with popliteal cysts, including osteoarthritis (81%), medial meniscus tear (68%), loose body (38%), edema (35%), and patellofemoral cartilage injury (30%). Sansone and De Ponti^35^ reported that medial meniscus injury is key to the formation of popliteal cysts. In their study, 84% of patients with popliteal cysts had medial meniscus lesions. Martí‐Bonmatí *et al*.^37^ examined the incidence of popliteal cysts by MRI and identified 145 popliteal cysts in 382 knees. There was a very significant correlation between effusion of knee cavity and popliteal cysts (*P* = 0.02) and between meniscus tear and popliteal cysts (*P* = 0.01). In this study, the most common intra‐articular pathology detected under arthroscopy was medial meniscus injury (69.6%).

In addition, valve mechanism is also a key factor in the persistence of popliteal cyst. Kim *et al*.^8^ have shown that a valvular mechanism exists between popliteal cysts and the articular cavity, which results in continuous unidirectional flow between the posteromedial compartment and the gastrocnemio‐semimembranosus bursa. The unidirectional communication portal should be corrected into two‐way flow during surgery. Many scholars have reported that arthroscopic expansion of the valvular mechanism is sufficient. Sansone and De Ponti^35^ described arthroscopic treatment of the associated intra‐articular pathology using the anteromedial approach, which corrects the valve mechanism by removing the posterior horn of the medial meniscus without removing the posterior capsule folds of the cyst. In a technical report, Takahashi and Nagano^38^ corrected the valvular mechanism by excising the “slit structure” through a posteromedial portal. Ahn *et al*.^30^ performed arthroscopy to correct intra‐articular lesions and excise the capsular fold through the posteromedial portal and reported satisfactory outcomes in 94% of patients during a mean follow‐up period of 36 months. In our study, the communication ports observed by MRI before operation were confirmed by arthroscopy, which were enlarged at least 5 mm.

### Arthroscopic Treatment With or Without Cystectomy

At present, proper treatment of the cyst wall has brought new challenges to the arthroscopic treatment of popliteal cysts. Interestingly, there are currently few controlled studies on cyst wall resection and preservation^39^. Therefore, whether removal of the cyst wall during surgery is necessary and the relationship between the residual cyst wall and recurrence of postoperative cysts remain controversial topics. Kongmalai and Chernchujit^24^ found that the walls of popliteal cysts are comprised of histologically thickened hyaloid tissue that does not contain any synovial fluid secreting cells. The authors believe that the cyst wall is simply a container for storing cyst fluid and does not produce synovial fluid. Therefore, it is not necessary to completely remove the cyst wall. Other researchers also support this view^40^, ^41^. Pankaj *et al*.^42^ performed arthroscopic decompression on 12 patients with popliteal cysts and retained the cyst wall. During an average follow‐up period of 24 months, only one patient showed no clinical improvement. Sansone and De Ponti^35^ reported a 95% success rate when correcting the valve mechanism and intra‐articular lesions without removing the cyst wall. Ko and Ahn^33^ retained part of the cyst wall structure in 14 cases, and observed no recurrence during postoperative follow‐up.

Numerous researchers prefer complete cyst wall resection. Chen *et al*.^43^ performed a resection of the cyst wall through a high posteromedial portal, and observed that most patients experienced significant improvement in clinical symptoms, with only one relapse. Ahn *et al*.^30^ introduced an improved arthroscopic technique, which used an additional portal known as the “posteromedial cystic portal” to perform a complete cystectomy on 24 patients with fibrous structures in the cyst. Follow‐up MRI studies showed that the cysts in all patients had disappeared or shrunk. In the most recent study, Gu *et al*.^26^ used double posteromedial portal to remove the cyst wall, and obtained satisfactory treatment results with no recurrence. The author believed that complete removal of the cyst wall combined with correcting the pathology and one‐way valve mechanism could reduce the possibility of recurrence.

### Possible Risks and Countermeasures During Surgery

To our knowledge, resection of the cyst wall increases operation time and the possibility of concomitant injury. The popliteal neurovascular bundle, which is usually on the outer side of the cyst, is the main neurovascular structure at risk during surgery. Large cysts often compress the adjacent popliteal artery and vein, resulting in slight displacement of blood vessels. Therefore, when using a shaver to remove the inner cyst wall, light suction should be applied to avoid drawing blood vessels and nerves into the shaver, especially when the cyst has extended to the posterolateral side. In addition, removal of the cyst wall requires the confirmation of an additional posteromedial portal, which is the accumulation of experience and puts forward higher surgical requirements for surgeons.

### Surgical Procedures for Different Types of Popliteal Cysts

Based on our experience, when there is a septum in the capsule, we recommend that experienced surgeons plane the cyst wall to reduce the potential for recurrence caused by the septum^32^. However, less experienced surgeons should pay more attention to complications and removal of the cyst wall should be of secondary concern. When the cyst is a single structure, it is sufficient to expand the communication port^44^ and relieve the internal pressure within the cyst. We believe that the symptoms caused by a cyst have nothing to do with its size, but reflect the location of the cyst and the pressure on the surrounding tissue. After fully expanding the communication port decompression, few patients will experience recurring symptoms, so there is no need for additional treatment of the cyst wall.

### Limitations of the Study

Our research has several limitations. First, we have designed a retrospective control study, but the inherent limitation of retrospective study should be considered. Second, the follow‐up period was relatively short and cysts could recur at a later time. In addition, our small sample size may limit the statistical power of the study. Due to these factors, the conclusion reached may be biased. These limitations need to be addressed by conducting a multi‐center, long‐term follow‐up randomized control study with a large sample population in the future.

### Conclusion

In conclusion, for unicameral cysts, resection of the cyst wall is not recommended, because it does not benefit in terms of clinical outcomes and recurrence rate compared with retaining the cyst wall; on the contrary, it increases the operation time and the probability of complications.
